# Goffin's cockatoos discriminate objects based on weight alone

**DOI:** 10.1098/rsbl.2021.0250

**Published:** 2021-09-08

**Authors:** Poppy J. Lambert, Alexandra Stiegler, Theresa Rössler, Megan L. Lambert, Alice M. I. Auersperg

**Affiliations:** ^1^ Comparative Cognition Unit, Messerli Research Institute, University of Veterinary Medicine Vienna, University of Vienna, Medical University of Vienna, Vienna, Austria; ^2^ Department of Behavioural Biology, University of Vienna, Althanstraße 14, 1090, Vienna, Austria; ^3^ Department of Cognitive Biology, University of Vienna, Althanstraße 14, 1090, Vienna, Austria

**Keywords:** weight discrimination, discrimination learning, physical cognition, parrot cognition

## Abstract

Paying attention to weight is important when deciding upon an object's efficacy or value in various contexts (e.g. tool use, foraging). Proprioceptive discrimination learning, with objects that differ only in weight, has so far been investigated almost exclusively in primate species. Here, we show that while Goffin's cockatoos learn faster when additional colour cues are used, they can also quickly learn to discriminate between objects on the basis of their weight alone. Ultimately, the birds learned to discriminate between visually identical objects on the basis of weight much faster than primates, although methodological differences between tasks should be considered.

## 1. Introduction

Decisions based upon an object's weight can improve the efficiency and success of natural behaviours. During extractive foraging, for example, assessing weight is one way to infer the nutritional value of a food resource. Cebus monkeys will preferentially choose a heavier nut [1], and food-caching passerines discard more light seeds than heavy ones [2,3]. Assessing weight may also be critical for optimizing instrumental problem solving; for instance, chimpanzees and Cebus monkeys select stone hammers for nut cracking based upon their weights [4–6].

To date, research on weight discrimination learning has largely focused on primates, which have shown surprising difficulties with the tasks presented to them. Long-tailed macaques, spider monkeys and a capuchin monkey required hundreds of trials (medians of 586, 330 and 321, respectively) and chimpanzees a median of 1100 trials to learn to consistently differentiate between visually identical light and heavy objects [7,8]. In a sorting task, where subjects needed to place visually identical objects in coloured trays according to weight, chimpanzees took an average of nearly 900 trials to reach criterion [9]. When presented with 12 identical objects, from which subjects needed to exchange the correct six (light or heavy) with the experimenter, less than half of the apes (orangutans, gorillas and bonobos) tested reached criterion, requiring a median of 331 exchanges to do so [10].

Comparable research on non-primate animals is still lacking. However, some corvids and parrots have shown that they are able to discriminate weighted objects [11–13]. For example, New Caledonian crows learnt to choose either heavy or light objects, which all differed from each other in colour and shape, and transferred the rule to new, visually novel, objects [11]. To achieve a more valid comparison with the abilities of primate species, it is necessary to test if and when an avian model can discriminate between two objects based on weight alone (without additional visual differences between objects). The question of weight-related cognition in birds and how it relates to that of primates is interesting, given the largely differing lifestyles of the two groups. It is likely that transporting items in flight, rather than carrying them on the ground, is more energetically demanding and thus requires greater sensitivity to the property of weight; a notable difference between the performance of birds and primates on weight-based discrimination tasks might reflect an interplay between environment and cognition.

We investigated weight discrimination in the Goffin's cockatoo, *Cacatua goffiniana* (Goffin hereafter). The Goffin provides an ideal avian model: in the wild, they acquire a substantive part of their diet through extractive foraging [14] and they have demonstrated sophisticated object and tool use, and tool manufacture skills, in the laboratory (e.g. [15–17]), for which proprioceptive feedback is also important (e.g. [18]).

We asked whether the Goffins can discriminate visually identical objects based on weight, and whether such discriminations are aided when subjects are given previous experience discriminating versions of the objects which additionally differ in colour. We predicted that the Goffins would be able to discriminate the weighted objects when they had no visual differences. Furthermore, if subjects attended to weight even when objects could be discriminated by colour, we predicted that they would then perform better with visually identical versions than subjects without this experience.

## 2. Material and methods

### (a) Subjects and testing groups

Sixteen Goffins (13 adults, M:8, F:5; and 3 sub-adults, M:1, F:2) participated. Subjects were randomly assigned to one of two groups (‘Pre-experience’ or ‘Test-only’; *n* = 8 for both) and to heavy or light as their rewarded object (balanced within groups). Subjects in the Pre-experience group were further assigned a rewarded colour (for a task where objects differed in both colour and weight), and the colour-weight combinations of the two objects remained constant. The two colours were counterbalanced against the two weights across the Pre-experience subjects. All these steps were completed controlling for sex and age as far as possible (see the electronic supplementary material, table S1). See the electronic supplementary material for details on subject housing and experience.

### (b) Apparatus

The objects were spheres 2 cm in diameter, made out of baked modelling clay (Fimo^®^). The heavy objects (23 g) featured a fishing weight inside, and the light objects (4 g) featured a compressed cotton ball. Objects in the weight-only condition were grey; objects in the colour-weight condition were purple or blue. Two transparent plastic dishes 10 cm in diameter and a third black dish with a lip and raised side (3 cm), 9.5 cm in diameter, were also used.

### (c) Procedure

**Figure 1.**
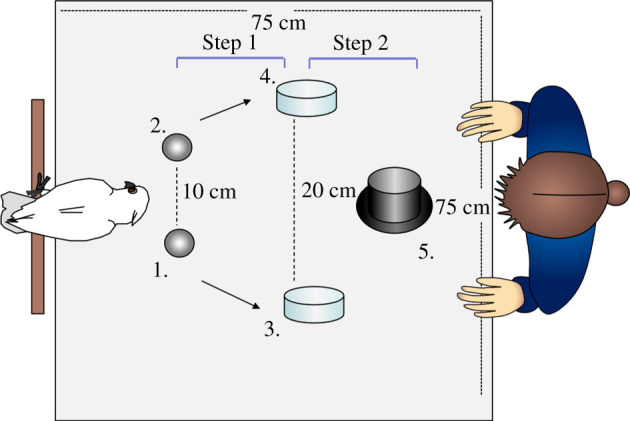
The order (1 to 5) of object placement (1 and 2 by the experimenter, 3 to 5 by the subject) in a trial. Arrows show the object movement by the subject. Distances are indicated by the dotted lines and recorded in cm.

The Pre-experience group was first given the discrimination task with objects which differed in colour and weight (colour-weight task) and later were tested with objects only differing in weight (weight-only task). The Test-only group only ever received the weight-only task. See the electronic supplementary material, figure S1 for a depiction of the testing regime. The procedure for a trial was the same for both tasks and is depicted in figure 1.

Before each trial, the experimenter placed the objects and transparent dishes on the table. In Step 1 of the trial, to give the subjects the opportunity to perceive the weight of both objects, the subject was asked by the experimenter (by using a familiar command, ‘give me’ and pointing) to place both objects, in turn, into the two adjacent transparent dishes. Subjects were given a low-value reward (sunflower seed) each time. In Step 2, the experimenter positioned and pointed at the black dish. If the subject placed the correct object, they received a high-value reward (cashew piece). The experimenter wore mirrored sunglasses and avoided lateral head movements. See the electronic supplementary material for further details of our procedure.

Sessions usually consisted of 10 trials. One session was given to a subject per testing day. Subjects were tested in the colour-weight task (Pre-experience group) and weight-only task (Test-only group), until they offered the correct object in eight consecutive trials, or nine in total, within a block of 10 (one Pre-experience subject was erroneously given one session beyond this point). There was a maximum of 10 sessions in the weight-only task if the criterion was not met, but no limit in the colour-weight task, given the predicted ease of discrimination. After meeting the criterion, subjects in both groups were given five weight-only sessions (the sessions of two Pre-experience subjects were interrupted; see the electronic supplementary material). All trials were video-recorded.

### (d) Analysis

We ran six generalized linear mixed models. To address our main research question—whether Goffins learned to discriminate objects on the basis of weight alone—we ran one model using data from the five sessions given after subjects met the criterion. With a second model, we tested whether Pre-experience subjects met criterion quicker on the colour-weight task than Test-only subjects on the weight-only task, using the sessions until criterion. Third, to investigate whether the Pre-experience group was more successful (i.e. made more correct choices) than the Test-only group, we compared all sessions of both groups in the weight-only task. The next two models addressed *post hoc* questions, focused on switching behaviour (putting down the first object picked up in Step 2 to pick up the other). To assess whether this behaviour was functional, for example whether it served to correct incorrect choices (upon receiving proprioceptive information again), we asked whether switching behaviour predicted probability of success, and whether the first choice in Step 2 (correct or incorrect) predicted whether the subject proceeded to switch. If subjects dropped an object (this may or may not be followed by a switch), the sound created differed between the heavy and light versions. To ensure that subjects could discriminate by weight, we ran our first model including only the trials (462 of 650 post-criterion trials) without drops. See the electronic supplementary material for further information on model structure, output and validation of model assumptions.

## 3. Results

The Test-only group required a mean of 60.6 trials (range: 30 to 88) to reach criterion, although one subject did not reach criterion in the given time (subject's data not included above). After criterion, the seven Test-only subjects maintained a high level of performance: 90.9% of choices were correct (subject range 76–98%; 6/7 subjects achieved 90% or higher correct). The Pre-experience group (*n* = 8) required a mean of 40.8 trials (range: 20–70) to meet criterion in the colour-weight task. These subjects (excluding the data of two subjects due to interrupted sessions; see the electronic supplementary material) achieved 77.6% correct choices overall in the five weight-only sessions (subject range: 60–92%).

**Figure 2.**
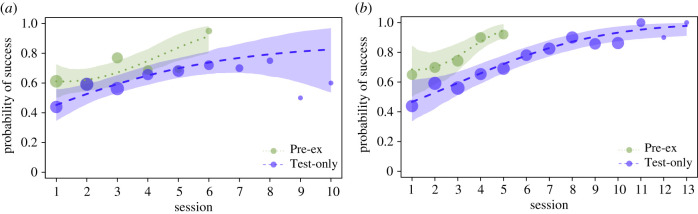
Influence of the experimental group on probability of success, for (*a*) the Pre-experience group in the colour-weight task and Test-only group in the weight-only task (until criterion) and (*b*) both groups in all their sessions of the weight-only task.

Analysing sessions after criterion, we found that subjects had learned to discriminate between the objects on the basis of weight (model 1: post-criterion sessions only; intercept estimate: 1.678, s.e. ± 0.230, *z* = 7.287, *p* < 0.001), with a predicted probability of 0.843 of choosing correctly. Our finding was similar when trials with drops were excluded (intercept estimate: 1.472, s.e. ± 0.266, *z* = 5.523, *p* < 0.001). There was a significant difference between groups in their performance on their respective (first) tasks: Pre-experience subjects performed better on the colour-weight task than the Test-only subjects on the weight-only task (figure 2*a*) (model 2: all sessions until reaching criterion; full-null model comparison: *χ*^2^ = 19.364, d.f. = 6, *p* = 0.004; group estimate: −0.624, s.e. ± 0.197, *z* = −3.167, *p* = 0.002). The Pre-experience group was also more successful on the weight-only task than the Test-only subjects (figure 2*b*) (model 3: test sessions only; full-null model comparison: *χ*^2^ = 18.768, d.f. = 6, *p* = 0.005; group estimate: −1.110, s.e. ± 0.272, z = −4.086, *p* < 0.001).

Although switching behaviour only occurred in 237 of the 1640 trials (212 were weight-only trials; see the electronic supplementary material, table S3 for a further breakdown according to task, number and directions of switches), we found that switching predicted success (model 4: full-null model comparison: *χ*^2^ = 18.669, d.f. = 2, *p* < 0.001; switch estimate: 1.806, s.e. ± 0.438, *z* = 4.119, *p* < 0.001). Additionally, first choice predicted subsequent switches—a subject was more likely to switch if they initially picked up the incorrect object (model 5: full-null model comparison: *χ*^2^ = 40.177, d.f. = 2, *p* < 0.001; first object estimate: 9.270, s.e. ± 1.511, *z* = 6.136, *p* < 0.001). Estimates from all models with the associated minimum, maximum and confidence interval values can be found in the electronic supplementary material, tables S4–S17.

## 4. Discussion

Our findings confirm our prediction that Goffins are able to discriminate between visually identical objects of different weights. On the weight-only task, the Pre-experience group was more likely to be correct than the Test-only group. It is thus likely that subjects paid some attention to object weight in the colour-weight task. This mirrors findings of Lambert *et al.* [13] for kea and New Caledonian crows, where some subjects learnt about object weight when there was also a stable colour difference between light and heavy objects.

Notably, the Goffins reached criterion (7/8 subjects) in the weight-only task after an average of 60.6 trials. This is in stark contrast to results from apes where subjects reached criterion after a median of 331 ‘exchanges’ [10] or an average of 895.2 trials [9]. The chance probability of meeting the criterion in these studies was 0.001 [10] or less than 0.0000001 [9], whereas in ours, it was 0.0039 (8/8) or 0.0098 (9/10). However, the seven Test-only subjects that met our criterion also reached a performance akin to the criterion used in [10] (either 10/10 or 13/14 (spanning blocks) correct, chance *p* < 0.00098) at an average trial number of 69, and six birds achieved the criterion used in [9] (45/50), at an average trial number of 101.7. Our results suggest that some bird species may learn to discriminate between visually identical objects based on weight faster than primates. If so, this might reflect a higher sensitivity to weight. The origin of this could lie in the importance of ‘weight-related signals’ for airborne species when transporting and using materials [9]. However, in any comparison between species, the weights (and, thus, the likely salience) of objects relative to subject weight should be considered as a potential contributing factor to the speed at which individuals master a weight discrimination task. Note that the near 20 g difference between our objects represented 6–8% of subject body weight, a higher proportion than in primate studies [7–10]. For example, the 300 g difference between objects in [10] was around 0.0007% of the average species body weight of individuals meeting the criterion.

We found that subjects sometimes switched to a second object after they picked up their first in the trial's choice phase. We conducted *post hoc* analyses which revealed that subjects were more likely to switch when they began with the incorrect object, and that switching behaviour increased the success rate. Dropping produced a sound cue which differed between light and heavy objects, but subjects did not require this information to discriminate between the visually identical versions: their performance remained significantly above chance when trials with drops were not considered within the analysis. However, sound cues may be an important discriminating feature for birds feeding on small food items differing in weight [3].

Studies investigating weight discrimination are still rare and are mostly limited to large-brained species (e.g. [9,11,12,19,20]). Making comparisons between different animal groups is challenging, given that relative strength, and perhaps even morphology, will affect how a species perceives weight cues. Nevertheless, we believe undertaking studies on other animal groups could still be revealing. Our findings suggest greater sensitivity to weight in a bird versus higher primates, which highlights the importance of considering how ecological challenges faced by a species may differently shape cognitive abilities.

## Data accessibility

The data are provided in the electronic supplementary material [21].
